# Neonatal survival interventions in humanitarian emergencies: a survey of current practices and programs

**DOI:** 10.1186/1752-1505-6-2

**Published:** 2012-07-23

**Authors:** Jennifer O Lam, Ribka Amsalu, Kate Kerber, Joy E Lawn, Basia Tomczyk, Nadine Cornier, Alma Adler, Anne Golaz, William J Moss

**Affiliations:** 1 Bloomberg School of Public Health, Johns Hopkins University, Baltimore, MD, USA; 2 Save the Children, Washington, DC, USA; 3 Saving Newborn Lives, Save the Children, Cape Town, South Africa; 4 Centers for Disease Control and Prevention, Atlanta, GA, USA; 5 United Nations High Commissioner for Refugees, Geneva, Switzerland; 6 London School of Hygiene and Tropical Medicine, London, United Kingdom; 7 UNICEF, New York, NY, USA

## Abstract

**Background:**

Neonatal deaths account for over 40% of all deaths in children younger than five years of age and neonatal mortality rates are highest in areas affected by humanitarian emergencies. Of the ten countries with the highest neonatal mortality rates globally, six are currently or recently affected by a humanitarian emergency. Yet, little is known about newborn care in crisis settings. Understanding current policies and practices for the care of newborns used by humanitarian aid organizations will inform efforts to improve care in these challenging settings.

**Methods:**

Between August 18 and September 25, 2009, 56 respondents that work in humanitarian emergencies completed a web-based survey either in English or French. A snow ball sampling technique was used to identify organizations that provide health services during humanitarian emergencies to gather information on current practices for maternal and newborn care in these settings. Information was collected about continuum-of-care services for maternal, newborn and child health, referral services, training and capacity development, health information systems, policies and guidelines, and organizational priorities. Data were entered into MS Excel and frequencies and percentages were calculated.

**Results:**

The majority of responding organizations reported implementing components of neonatal and maternal health interventions. However, multiple barriers exist in providing comprehensive care, including: funding shortages (63.3%), gaps in training (51.0%) and staff shortages and turnover (44.9%).

**Conclusions:**

Neonatal care is provided by most of the responding humanitarian organizations; however, the quality, breadth and consistency of this care are limited.

## Background

Humanitarian emergencies are acute or chronic situations of conflict, war or civil disturbance, natural disasters, food insecurity or other crises that affect large civilian populations and result in significant excess mortality [1]. These crises are often characterized by the collapse of basic health services as well as local and national infrastructure, resulting in the need for international assistance and aid by humanitarian organizations [1]. Recent examples include the conflicts in Sudan and in the Democratic Republic of the Congo, the 2010 earthquake in Haiti, and the 2010 floods in Pakistan, all of which resulted in massive population displacement, distress and casualty. In addition to high fatality rates due to direct impacts of the disaster, a humanitarian crisis often leads to indirect and prolonged effects on population health that result in increased mortality and poor health outcomes.

Mothers and children are disproportionately affected and under-five mortality rates can be several-fold higher during crises [1,2]. In particular, newborns younger than 28 days old have the highest risk of mortality [3]. Of the ten countries with the highest neonatal mortality rates, six – Afghanistan, Central African Republic, Democratic Republic of the Congo, Pakistan, Sierra Leone and Somalia – are currently or recently affected by humanitarian emergencies [3]. Consensus is lacking regarding programmatic priorities for care of mothers and newborns, and this issue is related to a knowledge gap about the direct causes of deaths and the level of provision of newborn care services in these settings. Few mortality surveys in emergency settings accurately capture rates and causes of death in the neonatal period but those that have captured neonatal outcomes find the burden to be high. One survey of pregnancy outcomes among Burundian refugees in Tanzania in 1998 found that neonatal and maternal deaths accounted for 16% of deaths among the total population [4], and a study of Afghan refugees in Pakistan in 1999 and 2000 determined that neonatal mortality accounted for 19% of all deaths and was the single largest “cause” of death [5].

Increasing our understanding of the provision of neonatal care during humanitarian emergencies will provide useful information to guide future service delivery. We report the results of a survey of governmental and non-governmental organizations that provide primary healthcare in humanitarian emergencies to better understand current policies and practices for newborn care at the primary care level in emergency settings and identify factors that affect the implementation and maintenance of interventions to reduce neonatal morbidity and mortality. The key questions we sought to answer were: (1) what is the current use and promotion of evidence-based neonatal and maternal health interventions in a continuum of care approach; (2) what are the barriers to implementation of interventions; and; (3) do organizations have policies and guidelines for neonatal and maternal health care.

## Methods

A web-based survey was conducted to collect information on maternal and neonatal care services in humanitarian emergencies and to identify barriers to program implementation. Agencies that work in neonatal and maternal health within humanitarian emergencies were asked to participate and select one respondent per agency. Respondents were surveyed about their organization’s current practices for maternal and newborn care, specifically: 1) antenatal care; 2) childbirth services; 3) preventive and curative newborn care; 4) postnatal care for mothers and newborns; 5) maternal and child health services; 6) referral services; 7) training experiences and needs; 8) policies and guidelines; 9) information and data collection; 10) barriers to providing comprehensive maternal, newborn and child healthcare in emergency settings; and 11) organizational priorities. The survey consisted of 69 multiple-choice and short-answer questions.

A list was created that included the key partner organizations likely to provide neonatal and maternal health in humanitarian emergencies. A non-random, “snow-ball” sampling technique was used, in which key respondents from these partner organizations were identified and asked to complete the survey. Program managers were targeted as the primary respondents. The respondents included both persons at agency headquarters as well as country-based respondents. Respondents were asked to answer questions based on the location where their organization’s services were provided, regardless of whether they were stationed at headquarters or at an in-country site. Each country program within an organization was permitted to submit only one response, but individuals working in the same location for different organizations completed separate surveys. Respondents were requested to forward the survey to colleagues with neonatal and maternal health experience. The survey was pilot tested by respondents at Save the Children, The UN Refugee Agency (UNHCR) and Médecins Sans Frontières. The final version was administered in English and French from August 18, 2009 to September 25, 2009.

## Results

### Respondents

The survey results are based on 56 respondents representing 27 organizations (Table 1) working in 34 countries and regions. The respondents represented 37 non-governmental organizations (NGOs), 17 United Nations organizations and 2 governmental organizations. Most respondents (79%) worked in primary care facilities at the time of the survey. The majority of respondents were program managers or technical advisors (66%); 23% served as health coordinators at their organization. Fifty-two percent (52%) of respondents received the survey directly from the study team and 48% had the survey forwarded to them by a colleague. Responses could not be obtained from several humanitarian organizations on the original distribution list, including the Women’s Commissions, Ipas, Jhpiego, Management Systems International, John Snow Incorporated, CARE, Medecins San Frontieres, Concern Worldwide and Action Against Hunger USA.

**Table 1:** Organizations responding to the survey

**Organization**	**Number of respondents**	**% of total respondents**
**Indicated country- or region-specific work**		
Save the Children	8	14
The United Nations Children’s Fund (UNICEF)	8	14
American Refugee Committee/ARC International	5	9
International Rescue Committee	4	7
United Nations Population Fund (UNFPA)	3	5
World Health Organization (WHO)	2	4
International Medical Corps	2	4
The UN Refugee Agency (UNHCR)	2	4
Association of Medical Doctors of Asia (AMDA) – Nepal	1	2
African Medical and Research Foundation – Ethiopia	1	2
Birzeit University	1	2
Center for Health Policy and Innovation	1	2
Centre for Operations Research and Training	1	2
Christian Community Healthcare Foundation/International Peace Commission	1	2
Cooperativa MLO	1	2
Cooperazione Internazionale (COOPI)	1	2
East Star	1	2
GTZ Kenya Country Program, Dadaab	1	2
International Committee of the Red Cross (ICRC)	1	2
Loving Friend	1	2
Mae Tao Clinic	1	2
Ministry of Health – Tuvalu	1	2
Nas Foundation	1	2
White Ribbon Alliance for Safe Motherhood	1	2
World Vision International	1	2
**Indicated global-level work**		
International Rescue Committee	1	2
Médecins du Monde	1	2
Merlin	1	2
UNFPA	1	2
WHO	1	2
**Total number of respondents**	**56**	**~100%**

### Antenatal care

**Figure 1:**
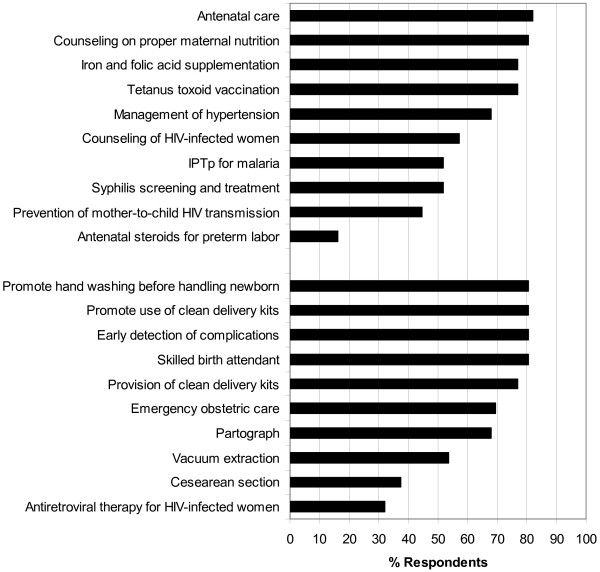
Provision of antenatal care (top) and childbirth services (bottom) in humanitarian emergency settings.

The majority (82.1%) of respondents indicated that antenatal care was provided by their organization, with most providing the following services: maternal nutrition counseling (80.4%), tetanus toxoid vaccination (76.8%), iron and folic acid supplementation (76.8%), identification and management of hypertension during pregnancy (67.9%), counseling of mothers with human immunodeficiency virus (HIV) infection (57.1%), syphilis screening and treatment (51.8%) and intermittent preventive treatment for malaria during pregnancy (IPTp) (51.8%) (Figure 1). However, fewer than half offered services to prevent mother-to-child transmission (PMTCT) of HIV (44.6%) or antenatal steroids for preterm labor (16.1%). Four respondents (7.1%) reported that none of the listed antenatal care services were provided at their organization.

### Childbirth services

A skilled birth attendant (SBA) was defined as an accredited health professional, such as a midwife, doctor or nurse, who was educated and trained in the skills needed to manage uncomplicated pregnancies, childbirth and the immediate postnatal period, and in the identification, management and referral of complications in women and newborns. To distinguish the presence of a SBA and provision of emergency obstetric care (EMOC), respondents could report the presence of a SBA at birth but no provision of EMOC, or the presence of a SBA at birth *and* provision of EMOC.

The majority of organizations (80.4%) had skilled attendants available to assist with births. The majority of respondents (80.4%) also claimed their organization promoted the use of clean delivery kits and 76.8% of respondents provided clean delivery kits (Figure 1). Hand-washing with soap and water prior to handling of the newborn was promoted by 80.4% of organizations. A lower proportion of organizations provided the following interventions during labor and delivery: emergency obstetric care (69.6%), partograph to monitor labor (67.9%), vacuum extraction (53.6%), and caesarian section (37.5%). Only one third (32.1%) offered antiretroviral therapy for mothers for PMTCT of HIV.

### Preventive newborn care

**Figure 2:**
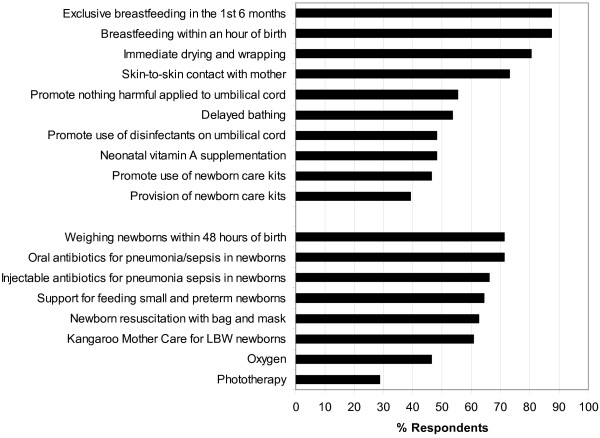
Provision of preventive (top) and therapeutic (bottom) newborn services in humanitarian emergency settings.

The majority of respondents reported provision of thermal care to newborns to prevent hypothermia, including immediate drying and wrapping of the infant (80.4%) and maternal skin-to-skin contact within 1–2 hours after birth (73.2%) (Figure 2). Fewer (53.6%) promoted a 6–24 hour delay after birth for bathing the newborn to prevent hypothermia. Most respondents (87.5%) reported that their organization promoted immediate breastfeeding within an hour of birth and exclusive breastfeeding for the first 6 months of life. Fewer than half of respondents reported the following preventive newborn care practices: promotion of the use of disinfectants for the umbilical cord (48.2%), promotion of the use of newborn care kits (46.4%) and provision of newborn care kits (39.3%). Newborn vitamin A supplementation coverage was also reported by less than half of respondents (48.2%), although this may not be policy in all settings.

### Therapeutic newborn care

More than half of respondents indicated that their organization weighed newborns within 48 hours of birth (71.4%), provided support for feeding small and preterm infants (64.3%), and/or promoted continuous and/or repeated skin-to-skin contact with the mother for low birth weight infants (73.2%) (Figure 2). The majority of organizations (62.5%) reported being able to perform newborn resuscitation with bag and mask, if necessary. However, fewer than half (46.4%) were able to provide oxygen. The majority of organizations reported they provided oral antibiotics (71.4%) or injectable antibiotics (66.1%) for infection management.

### Postnatal care services

Postnatal care was reported to be provided by 83.3% of surveyed organizations. Of these organizations, 65.2% provided home visits for the newborn within three days of birth as well as follow-up care within six weeks of birth. Immunizations were provided by 89.1% of sites which offered postnatal care.

### Child health services

Childhood immunizations were reported to be provided by 76.8% of organizations. The majority of programs surveyed also offered child vitamin A supplementation (73.2%), oral rehydration therapy (71.4%) and antibiotics for pneumonia (71.4%).

### Referral services

In settings of conflict or disaster, access to referral facilities may not be possible even if patient care is available at another site. One organization only referred patients that met eligibility guidelines, created to identify cases most likely to benefit from a referral. Newborns weighing less than 1.5 kg, for example, would not be eligible for referral because they are considered high risk. Several respondents described innovative strategies to document and improve referral systems for maternal and newborn health. One site makes use of a “Casa Materna”, or maternity waiting home, to bring women closer to the birthing site two to three weeks prior to their estimated due date.

### Training

Only 36.7% of respondents reported staff training on newborn care in the 12 months prior to the time of survey completion. Almost all respondents (91.8%) desired staff training on the management of neonatal complications, including neonatal sepsis. A large number of respondents also expressed a need for training on the care of low birth weight and preterm newborns (87.8%) and infection prevention (79.6%).

### Policies and guidelines

More than half of respondents (62.5%) reported having policies, and 66.7% reported having guidelines, on maternal health. The majority of policies and guidelines addressed the care of the mother during pregnancy and childbirth. Few policies or guidelines were reported to specifically address newborn and postnatal care. Of the organizations that reported having guidelines, those who worked in areas affected by humanitarian emergencies in the past decade were more likely to report having guidelines available for maternal health, child health and childbirth than for newborn care.

### Information systems and data collection for maternal and newborn health

A majority of respondents (72.9%) reported routinely collecting information on maternal and newborn care through health information systems (HIS). Twenty-three of 48 sites (47.9%) routinely collect information on maternal and newborn healthcare in emergencies in population-based household surveys separate from HIS. One program reported the use of maternal death audits and a geographical information system to collect information on maternal and newborn health outcomes and to lobby for increased resources.

### Barriers to maternal, newborn and child care

**Figure 3:**
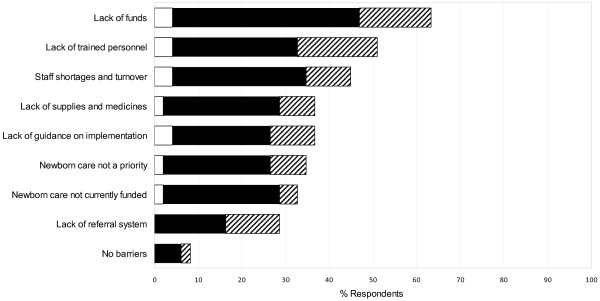
Barriers to providing newborn care services in humanitarian emergency settings, by organization type: open bar = governmental organization; solid bar = non-governmental organization; hatched bar = United Nations organization.

The most commonly reported barrier that organizations reported in delivering health care to mothers and newborns in humanitarian emergencies was insufficient funds (63.3%), followed by lack of trained personnel (51.0%) and personnel shortages (44.9%) (Figure 3). The types of barriers reported were similar across governmental, non-governmental and United Nations organizations. The likelihood of experiencing a particular barrier by organization type could not be assessed because NGOs were disproportionately represented.

### Health care priorities in humanitarian emergencies

Respondents were asked to rank provision of maternal, child and newborn health care, and control of disease outbreaks in humanitarian emergencies on a scale of 1 to 5, with 1 being the lowest priority and 5 being the highest priority. Maternal health was ranked the highest (mean rank 5), followed by control of disease outbreaks (mean rank 4.5). Child and newborn health ranked the lowest (mean rank 4.2 for both) as a healthcare priority during emergency situations. Although child and newborn health ranked the lowest relative to the other categories, the difference between each ranking was less than one.

## Discussion

To our knowledge, this is the first survey of experts and practitioners working in emergency settings addressing current practices for maternal and newborn care. Our survey included responses from many of the largest organizations working in these settings and from 34 countries. Of the 21 countries with serious political violence or warfare in the last decade, resulting in over 10,000 deaths, 52% were included in this survey [6]. Of the 18 countries affected in the last decade by a natural disaster, such as an earthquake, tsunami, heat wave or famine, resulting in over 5,000 deaths, 6 (33%) were included in this survey [7].These respondents may have been self-selected for those most interested in newborn survival; hence, our results likely reflect the range of care across programs addressing newborn survival, rather than represent all programs in humanitarian settings.

The majority of respondents reported implementation of a range of maternal, newborn and child health interventions, although there was greater emphasis on maternal health care services. Not all programs implemented a comprehensive package of the highest impact interventions such as neonatal warming, cord care and breastfeeding [8].

Reasons listed by respondents as factors that hindered the provision of a comprehensive set of interventions for newborns included the lack of funds, lack of trained personnel and shortage of medical supplies. The same barriers exist for the implementation and strengthening of healthcare services in many low-income countries, even in non-emergency settings. Pre-existing operational and financial barriers can pose additional challenges to humanitarian aid and can affect the ability to provide care broadly and efficiently in emergency situations. The addition of neonatal care services in settings with limited resources is most feasible if low-cost interventions that require minimal training, expertise and labor are implemented.

Promotion of home-based care practices, such as clean cord care, exclusive breastfeeding, and delayed bathing of the newborn to prevent hypothermia do not require technical equipment and can be provided by all programs working in humanitarian emergencies during routine pregnancy and postnatal visits. Notably, postnatal home visits were reportedly provided by 83.3% of organizations surveyed. However, the content and frequency of these visits are unclear, especially in cases of acute emergency. Additional training and education could improve coverage and quality of home-based interventions, building upon the health messages currently provided through home visits.

Research in relatively stable yet resource-limited settings has identified simple, low-cost interventions that are effective in improving neonatal survival and health outcomes [8-10]. These interventions include preventive care such as keeping the baby warm and breastfeeding, as well as prompt recognition and management of complications including bag-and-mask resuscitation [11], Kangaroo Mother Care for preterm babies in hospital settings [12], and treatment of neonatal infections [13]. These interventions are a part of a series of health system packages of routine childbirth and postnatal care which link to care before, during and after pregnancy and aim to address the major causes of neonatal morbidity and mortality.

Examining the breadth of newborn services offered in humanitarian emergencies can help identify areas for improvement as well as provide models for the implementation of these services in programs where the service is lacking. Antenatal care was offered by the majority (82.1%) of organizations surveyed. However, life-saving antenatal services, such as antenatal steroids during preterm labor, were provided infrequently (16.1%). Similarly, whereas over 80% of sites offered a postnatal visit, only two-thirds reported prioritizing the visit to take place within the first 3 days after birth, the time of highest risk to both mothers and newborns and the most crucial period to ensure optimal neonatal health [14]. Enabling organizations to broaden the breadth and type of services provided may reduce many of these missed opportunities.

Many respondents indicated the need for training and guidelines specifically relating to care of newborns in emergencies, based on evidence-based packages shown to be effective in stable settings. The development of training protocols and manuals specifically addressing the needs of newborns in humanitarian emergencies can be helpful in establishing standards of care across programs and organizations.

Many respondents identified the need for additional funds, a functional supply chain for equipment and drugs and properly trained personnel to provide services such as antenatal steroids and neonatal resuscitation, as well as more advanced clinical services such as caesarian section. Task shifting, a method of task distribution in which cadres of health care workers are trained to provide various services in different settings, may facilitate the delivery of intrapartum and neonatal care services in emergency settings when the number of highly trained staff are limited [15]. The formation of home-hospital linkages to ensure that women have access to the health care they need and the development of partnerships between organizations offering complementary services may also increase coverage of newborn health services [16].

Several organizations reported that limited access to transportation negatively impacted referral of pregnant women and neonates. Lack of security and inability to move freely, in addition to limited access to healthcare facilities, were also major impediments to referral services in humanitarian emergencies. However, some programs indicated that they have embraced task shifting and others have maintained strong referral systems, both of which could serve as models for other programs and settings.

The scope and level of newborn care services reported by the survey respondents was unexpectedly high. For example, fewer than 1 in 4 births in health care facilities in low-income countries may have access to trained providers and equipment for neonatal resuscitation [17]. However, the results of the survey do not reflect whether the services provided by surveyed organizations were easily accessible in a timely manner by pregnant women and their newborns and whether the quality of services provided was adequate. The high level of interest and reported provision of dedicated newborn care services may point to an additional opportunity to deliver high quality newborn care services in these settings, highlighting the need for up-to-date evidence-based protocols, guidelines and training.

Information for newborn health in emergency settings is exceedingly sparse. Indicators collected in these settings are not consistently those considered to be the most important for newborn survival. The coverage and quality of maternal and neonatal health services provided in emergency settings need to be tracked and evaluated, consistent with recommendations for non-emergency settings [18]. Tracking birth outcomes has been highlighted as a sensitive marker of a health system’s overall function [15]. Monitoring newborn health data may strengthen the case for increasing organizational and health system capacity.

## Limitations

The survey provided general information about the breadth of facility-based newborn health programs in humanitarian emergency settings but was limited by the respondent-driven sampling, small sample size and the potential bias resulting from the fact that respondents may have had a greater interest in newborn care than non-respondents. This bias could have resulted in an overestimate of newborn care services in humanitarian emergencies. The data are not representative of neonatal health interventions in all communities affected by humanitarian emergencies or of all organizations. In fact, several respondents indicated that the types and breadth of care they indicated may not be uniform across all sites and facilities within their organization. Additionally, the survey assessed the provision of services by primary care organizations, not the quality of services provided, the accessibility of the services to families in need nor the impact of services on neonatal outcomes in the local regions surveyed. The survey results are also subject to selection bias as those who opted to participate may be more engaged in newborn care.

## Conclusions

Improvements in newborn survival and health in emergency settings is increasingly recognized as a knowledge and action gap. Findings from this survey demonstrate that interventions to improve newborn health are provided in the context of humanitarian emergencies but, even from our self-selected respondents, it is clear there are limitations in the quality, breadth and consistency of neonatal care services during crisis situations. This assessment can provide a baseline to inform future work aimed at improving care at birth and newborn health outcomes in emergencies. We recommend more systematic review and consensus to inform the development of guidelines and training manuals. Implementation research is critical, especially regarding how to build upon existing structures for home visits, the use of task-shifting and effective referral networks. Humanitarian emergency situations provide many challenges but the time has come to address the needs of the most vulnerable.

## Abbreviations

HIS: health information system; HIV: human immunodeficiency virus; IPTp: intermittent preventive treatment for malaria during pregnancy; NGO: non-governmental organization; PMTCT: prevention of mother-to-child HIV transmission.

## Competing interests

The authors declare they have no competing interests.

## Authors’ contributions

JOL designed the survey instrument, conducted the survey, analyzed the survey results and drafted the manuscript; RA conceived of the study and participated in the design of the survey instrument, the interpretation of the findings and the writing of the manuscript; KK participated in the design of the survey instrument, the interpretation of the findings and the writing of the manuscript; JEL participated in the design of the survey instrument, the interpretation of the findings and the writing of the manuscript; BT participated in the design of the survey instrument, the interpretation of the findings and the writing of the manuscript; NC participated in the design of the survey instrument, the interpretation of the findings and the writing of the manuscript; AA participated in the design of the survey instrument, the interpretation of the findings and the writing of the manuscript; AG participated in the design of the survey instrument, the interpretation of the findings and the writing of the manuscript; WJM supervised the conduct and analysis of the survey and participated in the design of the survey instrument, the interpretation of the findings and the writing of the manuscript. All authors read and approved the final manuscript.
